# Biological and toxicological effects of non-dioxin-like PCBs

**DOI:** 10.1186/1751-0147-54-S1-S16

**Published:** 2012-02-24

**Authors:** Matti Viluksela, Leo TM van der Ven, Dieter Schrenk, Hellmuth Lilienthal, Patrik L Andersson, Krister Halldin, Helen Håkansson

**Affiliations:** 1 Department of Environmental Health, National Institute for Health and Welfare (THL), Kuopio, Finland; 2 Laboratory for Health Protection Research, National Institute of Public Health and the Environment (RIVM), Bilthoven, The Netherlands; 3 Food Chemistry and Toxicology, University of Kaiserslautern, Kaiserslautern, Germany; 4 Center of Toxicology, Institute for Prevention and Occupational Medicine (IPA), German Social Accident Insurance, Ruhr University of Bochum, Bochum, Germany; 5 Department of Chemistry, Umeå University, Umeå, Sweden; 6 Institute of Environmental Medicine, Karolinska Institutet, Stockholm, Sweden; 7 Department of Environmental Toxicology, Uppsala University, Uppsala, Sweden

## Background

Polychlorinated biphenyls (PCBs) are a group of 209 congeners with similar basic structure, but differing in the number of chlorines and chlorination pattern. They are potent, persistent and accumulative, and abundantly present in food and the environment. A group of 12 congeners has a dioxin-like planar structure and toxicological properties similar to dioxins and are therefore called dioxin-like PCBs (DL-PCBs). Their risk assessment is included in that of other DL compounds. The rest of the PCBs, with non-planar structure due to chlorine substitution at *ortho* position, have a different toxicological profile with possibly several different mechanisms. They are referred to as non-dioxin-like PCBs (NDL-PCBs), and their toxic effects have been, so far, poorly characterized because of contamination of several used NDL-PCBs batches with very potent DL impurities (EFSA, 2005: http://www.efsa.europa.eu/de/scdocs/doc/284.pdf). The aim of the ATHON (Assessing the Toxicity and Hazard of Non-dioxin-like PCBs Present in Food) project was to provide missing critical health hazard information, to clarify biological mechanisms underlying the various types of toxicity of NDL-PCBs and to evaluate these data from the risk assessment point-of-view.

## Materials and methods

High purity model compounds PCB180 (2,2’,3,4,4’,5,5’-heptachlorobiphenyl) or PCB52 (2,2’,5,5’-tetrachlorobiphenyl) were given at several dose-levels to young adult rats for 28 days, and to pregnant female rats from gestation day 7 until weaning. Toxic effects and induction of xenobiotic metabolising enzymes were studied at the end of treatment and in the offspring using haematology, clinical chemistry, biochemistry, molecular biology, histopathology, neurobehavioural testing and tissue PCB level analyses.

## Results

The cytochrome P450 (CYP) induction profile of both NDL-PCBs in liver was clearly different from that of DL compounds and characteristic of constitutive active androstane receptor (CAR) and pregnane X receptor (PXR) agonists. Neither of them was hepatotoxic. Both congeners caused reduced levels of circulating thyroid hormones T4 and T3 in adult animals and at lower exposure levels also in pregnant females and in the offspring. The likely mechanism for hypothyroidism is increased hepatic clearance due to induced UGT activity and displacement of thyroid hormones from their transport protein transthyretin. Changes in retinoid metabolism were observed both after adult and perinatal exposure. Neurobehavioural effects included altered open field behaviour in adult females (PCB180), impaired auditory function in male and female offspring (PCB180 and PCB 52) and altered sexually dimorphic behaviour in female offspring (PCB180).

## Conclusions

High purity PCB180 and PCB52 cause distinct pattern of effects, partly similar to and partly different from those of DL-PCBs. Risk characterization based on the observed liver and thyroid hormone effects and adipose tissue concentrations (this study and human data) suggests a margin of exposure for the adult general human population, which is several orders of magnitude for these individual NDL-PCB congeners.

